# Bioanalysis of doxorubicin aglycone metabolites in human plasma samples–implications for doxorubicin drug monitoring

**DOI:** 10.1038/s41598-020-75662-w

**Published:** 2020-10-29

**Authors:** Christian Siebel, Claudia Lanvers-Kaminsky, Gudrun Würthwein, Georg Hempel, Joachim Boos

**Affiliations:** 1 Department of Paediatric Haematology and Oncology, University Children’s Hospital Muenster, Albert-Schweitzer-Campus 1, Building A1, 48149 Muenster, Germany; 2 Department of Pharmaceutical and Medical Chemistry - Clinical Pharmacy, University of Muenster, Muenster, Germany

**Keywords:** Oncology, Chemotherapy

## Abstract

The widespread clinical use of the cytostatic doxorubicin together with the induction of chronic cardiomyopathy necessitates the conduct of further pharmacokinetic trials. Novel analytical technologies suitable for point-of-care applications can facilitate drug level analyses but might be prone to interferences from structurally similar compounds. Besides the alcohol metabolite doxorubicinol, aglycone metabolites of doxorubicin might affect its determination in plasma. To evaluate their analytical relevance, a validated HPLC method for the quantification of doxorubicin, doxorubicinol and four aglycones was used. The degradation pattern of doxorubicin in plasma under long-term storage was analysed with respect to the formation of aglycone products. In addition, overall 50 clinical samples obtained within the EPOC-MS-001-Doxo trial were analysed. Substantial degradation of doxorubicin in plasma occurred within a storage period of one year, but this did not lead to the formation of aglycones. In clinical samples, 7-deoxydoxorubicinolone was the major aglycone detectable in 35/50 samples and a concentration range of 1.0–12.7 µg L^−1^. If at all, the other aglycones were only determined in very low concentrations. Therefore, analytical interferences from aglycones seem to be unlikely with the exception of 7-deoxydoxorubicinolone whose concentration accounted for up to 65% of the doxorubicin concentration in the clinical samples analysed.

## Introduction

The anthracycline doxorubicin (DOX) is widely used in the treatment of solid and haematological malignancies. Its use is particularly important in the treatment of paediatric malignancies, such as acute leukaemia, lymphomas, neuroblastoma, osteosarcoma, Ewing’s sarcoma and nephroblastoma^1^. The induction of chronic cumulative dose-depending cardiotoxicity is a severe and common side effect, which is of great concern for childhood cancer survivors given their long life expectancy and the progressive nature of DOX-induced cardiomyopathy^2^.

With respect to its clinical relevance and severe side effects, bioanalysis of DOX within pharmacokinetic trials remains to be important, in particular in childhood cancer patients^3^. However, implementation of drug monitoring for DOX is complex as bioanalysis of DOX is prone to pre-analytical errors due to the fast sequestration of the drug from plasma in blood cells and extensive metabolism^4^. Point-of-care devices based on new analytical technologies could facilitate pharmacokinetic analyses by reducing preanalytical errors and allowing for a fast and reliable determination of drug levels using smallest sample volumes. Within the DiaChemo project, our working group is currently involved in the development of a novel miniaturised analytical device for the bedside drug monitoring of DOX and other chemotherapeutics (https://www.diachemo.eu/). Also, electrochemical or optical detection techniques have been applied in drug monitoring including DOX monitoring^5–8^. However, when determining drug levels in biological fluids, structurally similar drug metabolites and degradation products have to be taken into account as these might interfere with the determination of the parent drug molecule. Analytical techniques that do not rely on conventional chromatographic separation might be particularly affected by such interferences. Therefore, detailed knowledge of potential interfering substances is essential to ensure selectivity of the analytical method. With regard to DOX, the potential role of aglycone products for bioanalysis requires further characterisation.

**Figure 1:**
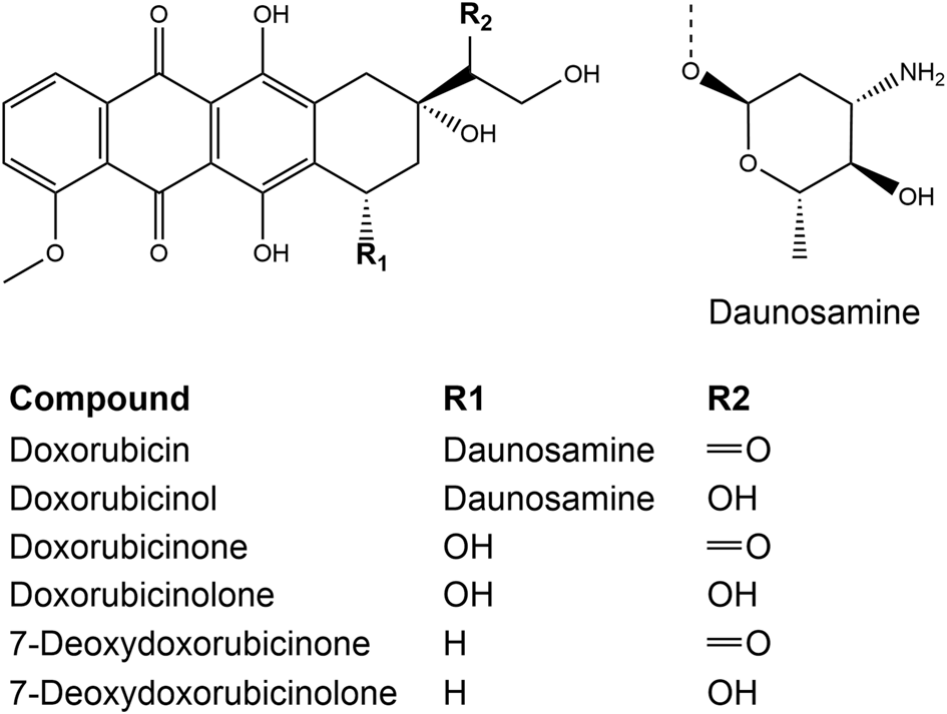
Structure of doxorubicin and its metabolites.

In vivo, DOX undergoes extensive metabolism. Doxorubicinol (DOXol) is the most common metabolite (Fig. 1) formed by two electron reduction of the C-13 carbonyl group to a hydroxyl group^9,10^. The reaction is catalysed by cytoplasmatic NADPH-dependent carbonyl and aldo–keto reductases in various tissues such as heart, liver, kidneys or red blood cells. DOXol is considerably less cytotoxic than the parent drug and is thought to contribute to its cardiotoxic effect^10–12^. Hydrolytic or reductive deglycosidation constitutes a minor metabolic pathway resulting in 7-hydroxy- or 7-deoxyaglycones (Fig. 1). Formation of the 7-hydroxyaglycones doxorubicinone and doxorubicinolone is catalysed by cytosolic NADPH-dependent glycosidases, whereas formation of the 7-deoxyaglycones 7-deoxydoxorubicinone and 7-deoxydoxorubicinolone is catalysed by microsomal oxidoreductases^12^. Aglycones do not exhibit cytotoxic activity but similar to DOXol they have been suggested to be cardiotoxic^12,13^. Subsequent metabolic steps involve *O*-demethylation, *O*-sulfation and *O*-β-glucuronidation^9^.

A number of studies investigated the pharmacokinetics of the aglycone metabolites. These studies provided inconclusive results regarding the type of aglycones that were detected in plasma of cancer patients as well as their expectable concentration range^14–18^. The purpose of the present analyses was to provide data from clinical plasma samples to allow for an accurate assessment of the potential relevance of aglycone metabolites for DOX drug monitoring. The formation of aglycone products in plasma samples under long term storage was also investigated.

## Methods

### Reference substances and chemicals

DOX was purchased from Sigma Aldrich Chemie GmbH (Munich, Germany). DOXol, doxorubicinone, doxorubicinolone, 7-deoxydoxorubicinone and 7-deoxydoxorubicinolone were purchased from Toronto Research Chemicals (Toronto, Canada). Stock solutions of the reference compounds were prepared in acetonitrile at a concentration of 20 mg L^−1^. The internal standard epirubicin was provided by Medac GmbH (Wedel, Germany) as a solution for infusion (2 g L^−1^). Acetonitrile, chloroform, ethanol, formic acid, disodium hydrogen phosphate and phosphoric acid were purchased in HPLC grade from various suppliers. Leucocyte-depleted fresh frozen plasma was provided by the Department of Transfusion Medicine of the University Hospital Muenster. Left-over plasma samples were used that were fully anonymized beforehand.

### Chromatography

For the determination of aglycones the HPLC method previously described by Kontny et al*.* was adapted^4^. Apart from DOX and DOXol the adapted HPLC method allows quantifying the aglycones doxorubicinone, doxorubicinolone, 7-deoxydoxorubicinone and 7-deoxydoxorubicinolone. Chromatographic separation of the aglycones was achieved by modifying the elution gradient of the original method. For sample preparation liquid–liquid extraction was performed with chloroform after adjustment of the pH to 8.5. To 100 µL of each sample 50 µL epirubicin was added as an internal standard. Overall 100 µL of phosphate buffer (pH 8.5) and 1000 µL of chloroform were added and the samples were mixed for 5 min on a rotation shaker. Subsequently, samples were centrifuged for 5 min at 4 °C and 20,000×*g*. The organic phase was transferred to a new polypropylene tube and evaporated to dryness under a stream of nitrogen at 35 °C. The residue was dissolved in 100 µL of a 0.1% formic acid in water: 0.1% formic acid in acetonitrile solution (75:25) and sonicated for 5 min before injection. An HPLC system equipped with a SIL-30AC autosampler, a RF-20Axs fluorescence detector and two LC-10ADvp pumps was used (Shimadzu, Duisburg, Germany). Analyses were run with a Purospher STAR RP-18 endcapped (5 µm) column (Merck KGaA, Darmstadt, Germany) and a mobile phase of 0.1% formic acid in water (A) and 0.1% formic acid in acetonitrile (B). The gradient for elution was composed of: 25–30% B over 7 min, 30–35% B over 11 min, 35–55% B over 7 min, 55–95% B over 0.5 min, 95% B for 5 min, 95–25% B over 0.5 min, 25% B for 5 min. Fluorescence of the analytes was excited at a wavelength of 488 nm and recorded at an emission wavelength of 555 nm. Separation of all analytes was achieved within 25 min.

The method was validated according to the European Medicines Agency (EMA) guideline on bioanalytical method validation^19^. In brief, the calibration range for DOX and DOXol was 2–1000 and 2–500 µg L^−1^. The aglycones could be determined in a concentration range from 1–100 µg L^−1^. Within- and between-run accuracy and precision were determined at four concentrations in five replicates each. Accuracy for all analytes was 97.6–115% (*within-run*) and 93.0–110% (*between-run*). The criterion for precision was met for all analytes with coefficients of variation ≤ 6.1% (*within-run*) and ≤ 8.6% (*between-run*). No interferences from endogenous components of the matrix or carry-over effects were detected. The aglycones proved to be stable in plasma when stored for 24 h at room temperature and for 12 weeks at − 20 °C or − 80 °C. Samples can be frozen and thawed for a minimum of three cycles. The extracted and evaporated samples could be stored for 24 h at − 20 °C. When stored in the autosampler, the extracted and re-dissolved samples were stable for at least 24 h at 4 °C. Stock solutions of the aglycone metabolites can be stored at room temperature for 24 h (see Supplementary Information S1 for detailed results of the method validation). Stability data for DOX and DOXol have been reported previously^4,20^.

### Determination of degradation products in plasma

The development of aglycones as products of degradation processes during long-term storage of DOX in plasma was investigated. Plasma was spiked with DOX at 25 µg L^−1^ and 500 µg L^−1^, respectively, and stored at 4 °C, − 20 °C and − 80 °C to reflect commonly used storage conditions. Three aliquots per concentration were immediately analysed for DOX and its metabolites and periodically within a period of about one year. In line with the EMA guideline quality control samples at three concentrations were analysed at the beginning and end of each analytical run.

### Determination of metabolites in clinical samples

DOX, DOXol and the aglycone metabolites were analysed in a subset of 50 clinical samples from overall 44 paediatric cancer patients obtained within the pharmacokinetic phase-II EPOC-MS-001-Doxo trial (EudraCT number 2009-011454-17)^21^. Reanalysis of stored patient samples was approved by the competent local ethics committee (reference number 2016-154-f-S). Within the EPOC-MS-001-Doxo trial samples were analysed for DOX and DOXol. For reanalysis, samples were selected based on the original measurement results such that the whole concentration range of DOX was covered. To account for potentially short plasma half-lives of the aglycone metabolites, samples were selected that were taken either during DOX infusion or up to 4.25 h after the end of infusion to ensure detection of the metabolites. At the time of analysis the samples have already been stored for 6–8 years at − 80 °C. As described above, quality controls where analysed with each analytical run.

### Ethics approval

Clinical samples were obtained within the EPOC-MS-001-Doxo trial (EudraCT number 2009-011454-17). This trial did directly address the pharmacokinetics of Doxorubicin in children. The trial was conducted in accordance with the Declaration of Helsinki, the International Conference on Harmonisation Good Clinical Practice Guidelines and applicable local regulatory requirements and laws. Written informed consent for participation in the EPOC-MS-001-Doxo trial including the analysis of Doxorubicin and metabolites was obtained from the patients’ parents or legal representatives. The use of stored clinical samples from the EPOC-MS-001-Doxo trial for the analyses described here was approved by the local ethics committee (ethics committee of the medical association Westfalen-Lippe and the University of Münster; reference number 2016-154-f-S).

## Results

### Determination of degradation products in plasma

**Figure 2:**
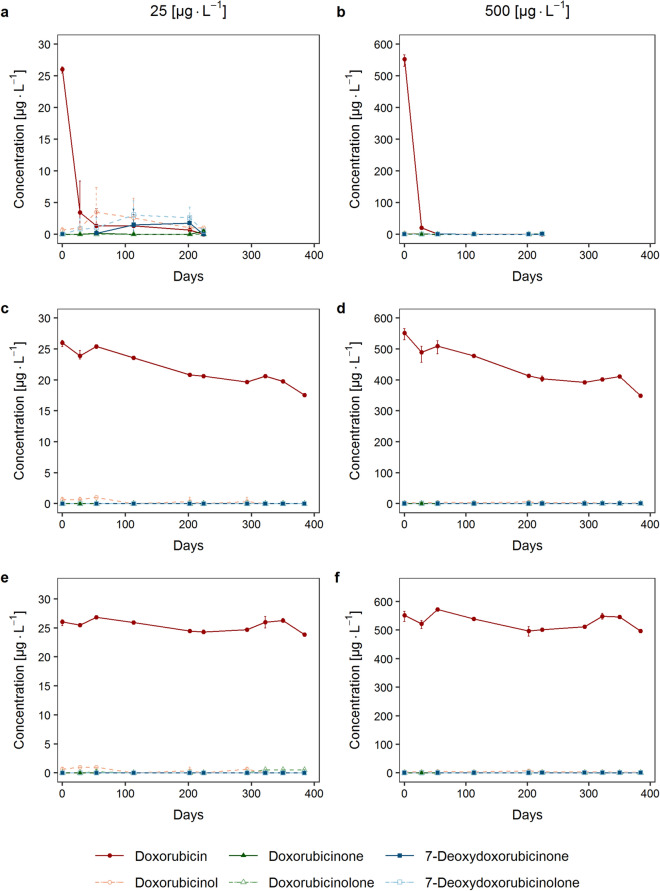
Concentration profile of doxorubicin and the metabolites doxorubicinol, doxorubicinone, doxorubicinolone, 7-deoxydoxorubicinone and 7-deoxydoxorubicinolone in plasma during storage. **a,b** samples stored at 4 °C; **c,d** samples stored at − 20 °C; **e,f** samples stored at − 80 °C. Plasma samples were spiked with doxorubicin to a concentration of either 25 µg L^−1^ or 500 µg L^−1^. At each time point, analyses were performed in triplicate. Data are presented as mean values and error bars indicate the minimum and maximum measurement value.

Upon storage of spiked plasma samples at 4 °C DOX degraded almost completely within a few weeks (Fig. 2). After four weeks the DOX concentration relative to the initial concentration was 13.3% (25 µg L^−1^) and 3.7% (500 µg L^−1^), respectively. At − 20 °C the concentration fell below 85% of the initial concentration after storage for four months. A reduction of the DOX concentration to 67.4% (25 µg L^−1^) and 63.2% (500 µg L^−1^) was observed after storage for about one year at − 20 °C. When stored at − 80 °C, DOX proved to be stable for at least one year (91.7% and 90.1%, respectively). The data on storage stability complement those reported previously^4,20^. Though considerable degradation occurred both at 4 °C and − 20 °C no concomitant increase in the concentration of any of the four aglycones was detected (Fig. 2).

### Determination of metabolites in clinical samples

**Figure 3:**
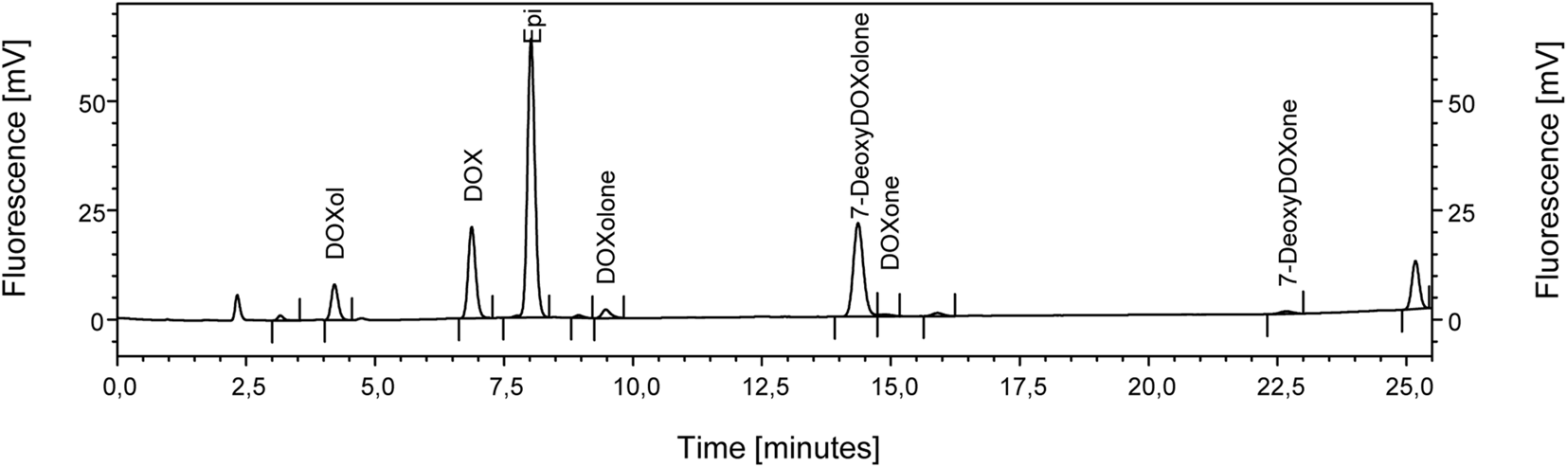
Representative chromatogram of a clinical sample from the EPOC-MS-001-Doxo trial. Epirubicin was added as an internal standard. 7-DeoxyDOXone, 7-deoxydoxorubicinone; 7-DeoxyDOXolone, 7-deoxydoxorubicinolone; DOX, doxorubicin; DOXol, doxorubicinol; DOXolone, doxorubicinolone; DOXone, doxorubicinone; Epi, epirubicin.

**Figure 4:**
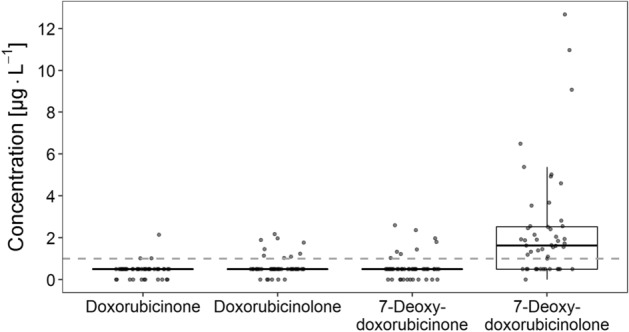
Plasma concentration of the aglycone metabolites in 50 patient samples obtained within the EPOC-MS-001-Doxo trial. The grey dashed line indicates the lower limit of quantification of the HPLC method of 1 µg L^−1^. For analysis, measurement values between the detection limit and the quantification limit were set to one half of the quantification limit (0.5 µg L^−1^).

Although all four aglycone metabolites were detected in patient samples obtained from the EPOC-MS-001-Doxo trial, large differences exist regarding the proportion of samples in which each metabolite was detectable (see Fig. 3 for a representative chromatogram of a clinical sample). 7-Deoxydoxorubicinolone was the major aglycone metabolite and was quantified in 35 out of the 50 samples. In these samples the concentration of 7-deoxydoxorubicinolone ranged from 1.0 to 12.7 µg L^−1^ (Fig. 4). In contrast, the aglycones doxorubicinone, doxorubicinolone and 7-deoxydoxorubicinone were only quantified in a few samples (doxorubicinone: 3/50 samples; doxorubicinolone: 9/50 samples; 7-deoxydoxorubicinone: 8/50 samples) and in very low concentrations (doxorubicinone: 1.0–2.1 µg L^−1^; doxorubicinolone: 1.0–2.2 µg L^−1^; 7-deoxydoxorubicinone: 1.0–2.6 µg L^−1^) (Fig. 4). Overall, in 13/50 samples none of the four aglycones was quantified.

Until reanalysis, the samples from the EPOC-MS-001-Doxo trial have been stored for 6–8 years at − 80 °C. Relevant degradation of DOX below 85% of the initially measured concentration value was observed in a subset of 14 samples which might have led to an increase in the concentration of aglycones. As indicated by the stability analysis described by Fig. 2, degradation of DOX during long-term storage does not lead to the formation of aglycones. Thus, aglycones quantified in the clinical samples can be considered to represent products of in vivo metabolism of DOX.

## Discussion

A point-of-care analytical platform for the detection of chemotherapeutics is currently under development within the DiaChemo project (https://www.diachemo.eu/). The technology is based on selective binding of drug molecules by functionalised gold nanoparticles and separation of drug-bound nanoparticles via a liquid crystal matrix^22,23^. Within the project DOX serves as a model substance. Various other non-chromatographic techniques have been employed for drug level analyses that are based on electrochemical or optical detection^5–8^. Point-of-care analytics that minimise preanalytical variability and reduce technical effort will substantially facilitate drug monitoring. In the case of DOX, paediatric cancer patients might particularly benefit from point-of-care analytics which offer the possibility to improve dosing strategies and potentially increase the safety of DOX administration in this vulnerable patient population^3^.

With regard to novel bioanalytical methods for DOX, pronounced plasma concentrations of the aglycones might interfere with the determination of DOX. Based on available data alone, the relevance of aglycones for DOX drug monitoring cannot be adequately defined. However, in our analysis the aglycones doxorubicinone, doxorubicinolone and 7-deoxydoxorubicinone were infrequently quantified in the samples from the EPOC-MS-001-Doxo trial and, if at all, in very low concentrations (doxorubicinone: 1.0–2.1 µg L^−1^; doxorubicinolone: 1.0–2.2 µg L^−1^; 7-deoxydoxorubicinone: 1.0–2.6 µg L^−1^) (Fig. 4). From an analytical point of view, these metabolites are presumably of little significance. The situation might be different for 7-deoxydoxorubicinolone which constituted the major aglycone metabolite in the clinical samples analysed here. Though the concentration of 7-deoxydoxorubicinolone was low in most of the samples (1.0–12.7 µg L^−1^), the relative content of this metabolite compared to the concentration of the parent drug DOX was 65% in one sample. Overall, in 5 out of the 35 samples in which 7-deoxydoxorubicinolone could be quantified the concentration relative to DOX exceeded 10%. Potential analytical interference from this metabolite has therefore to be taken into account when developing a point-of-care device for the quantification of DOX.

It has to be underlined that the clinical samples analysed here have been stored for 6–8 years from sampling until reanalysis. The use of stored samples raises two concerns. Firstly, relevant degradation of DOX was observed in 14 out of 50 samples and concomitant formation of aglycones might have been occurred during storage. However, we were not able to detect the formation of aglycones in plasma samples spiked with DOX when stored at 4 °C, − 20 °C or − 80 °C, though considerable degradation occurred at both 4 °C and − 20 °C (Fig. 2). This observation points to the formation of other decomposition products, possibly 6,8,11-trihydroxy-1-methoxy-5,12-naphthacenedione, which has been identified as a component in decomposition mixtures following degradation of DOX in aqueous solution^24^. Of note, our results differ from stability data obtained upon short-term storage of DOX in plasma at 37 °C or room temperature which indicate that 7-deoxyaglycones might be formed in consequence of the degradation of the parent drug molecule^11,25^. The discrepancies between these results and ours may arise from both the differences in the storage period as well as in the storage conditions. Nevertheless, the limited storage stability of DOX need to be considered when reanalysing stored study samples e.g. when used as quality controls. Secondly, degradation of the aglycones themselves might have occurred during storage and hence the concentrations measured in the samples might be artificially low. Within validation of the HPLC method stability of the aglycones has only been proven for 3 months (see Supplementary Information S1). However, the results of our analysis are in accordance to those of previous studies which reported substantially low concentrations of the aglycone metabolites^14–16^.

In contrast to the reports by Joerger et al*.* and Beijnen et al*.* who did not observe 7-deoxydoxorubicinolone^15,16^, this metabolite constituted the major aglycone in the subset of patient samples from the EPOC-MS-001-Doxo trial. Discrepancies between studies in the type of aglycones observed in plasma might be attributed to potential differences in DOX metabolism, the quantification limit of the analytical method and the small number of patients included in the majority of studies. In our analysis, 7-deoxydoxorubicinolone could not be quantified in 15 out of 50 samples (30%) and in 14 of those 15 samples measurement values were between the detection limit and the quantification limit. Likewise, Cummings et al*.* reported 7-deoxyaglycones to be present in about 60% of patients^18^. Hence, very low aglycone concentrations and small patient numbers hamper the detection of these metabolites. Exemplarily, only seven patients were included in the analysis by Joerger et al*.*^15^.

In conclusion, the data presented in this article confirm that aglycone metabolites are present in human plasma samples, but, with the exception of 7-deoxydoxorubicinolone, only very low concentrations were detected. Thus, our results support previous data that showed only low levels of the aglycones in plasma. In the subset of samples analysed, 7-deoxydoxorubicinolone was the major aglycone metabolite, which might be of relevance with regard to analytical interferences. Further, it seems to be unlikely that aglycones are formed as products of DOX degradation under storage conditions. The analyses underline the necessity to establish data on drug metabolites and degradation products and will help to avoid misinterpretation of doxorubicin drug monitoring results.

## Supplementary information

Supplementary Information

## Data Availability

The datasets analysed during the current study are available from the corresponding author on reasonable request.
