# Supplementary material for: Bioanalysis of doxorubicin aglycone metabolites in human plasma samples–implications for doxorubicin drug monitoring
Source: Sci Rep. 2020 Oct 29;10:18562. doi: 10.1038/s41598-020-75662-w (PMC7596548; doi:10.1038/s41598-020-75662-w)
Supplement: Supplementary file 1 — Supplementary Information [file 41598_2020_75662_MOESM1_ESM.pdf]

Supplementary Information S1

Bioanalysis of doxorubicin aglycone metabolites in human plasma samples – implications for doxorubicin drug monitoring

Christian Siebel, Claudia Lanvers-Kaminsky, Gudrun Würthwein, Georg Hempel, Joachim Boos

Correspondence: Joachim Boos; Department of Paediatric Haematology and Oncology; University Children's Hospital Muenster; Albert-Schweitzer-Campus 1; Building A1; 48149 Muenster. Email: Sekretariat.Boos@ukmuenster.de; Phone: +49 251 83-55657; Fax: +49 251 83-55740

Results of the HPLC method validation procedure

Method validation was performed according to the European Medicines Agency guideline on bioanalytical method validation [1].

Table of contents

I. Selectivity (figures S1, S2)
II. Carry-over (figure S3)
III. Calibration curve (table S1)
IV. Accuracy & precision (table S2)
V. Stability (tables S3 – S8)

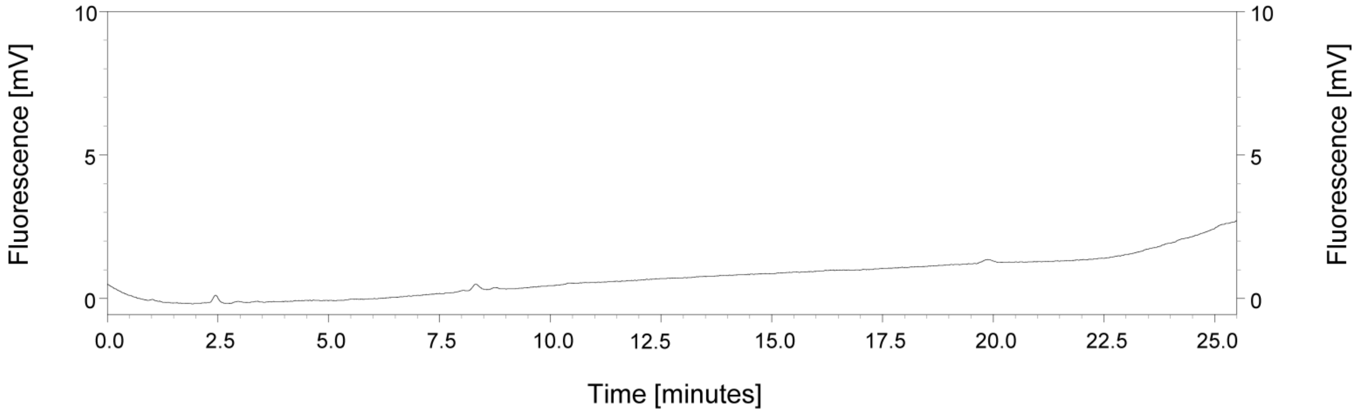

Figure S1: Illustrative chromatogram of an anthracycline-free plasma sample. Plasma samples from six individual donors were analysed.

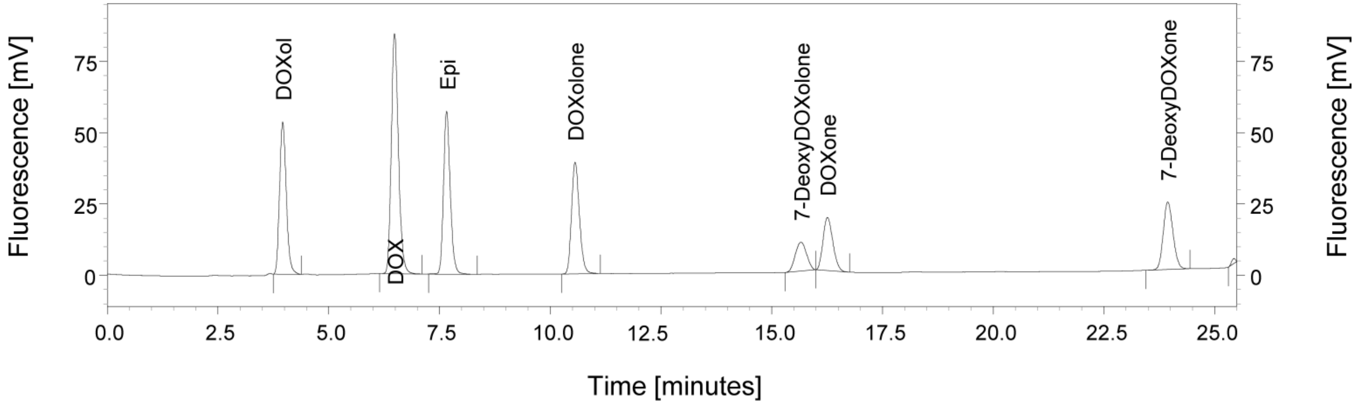

Figure S2: Illustrative chromatogram of a plasma sample spiked with all analytes and the internal standard epirubicin. Overall, six different plasma samples were analysed. Samples were spiked with doxorubicin (DOX) and doxorubicinol (DOXol) at a concentration of 100 µg·L^{-1}. The aglycones doxorubicinone (DOXone), doxorubicinolone (DOXolone), 7-deoxydoxorubicinone (7-DeoxyDOXone) and 7-deoxydoxorubicinolone (7-DeoxyDOXolone) were added to a concentration of 10 µg·L^{-1}. The internal standard epirubicin (Epi) was added to a concentration of 40 µg·L^{-1}.

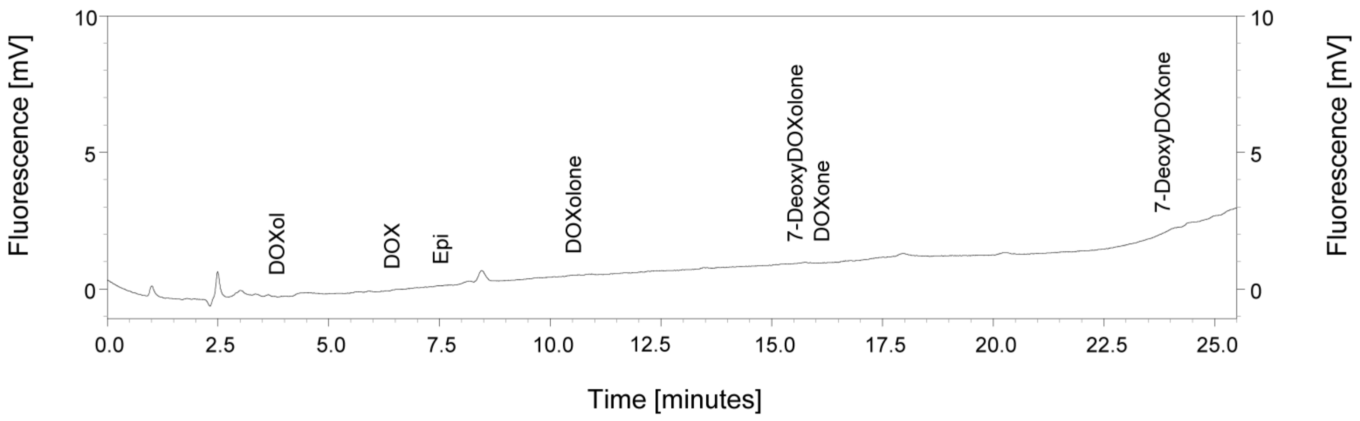

Figure S3: Illustrative chromatogram of a blank plasma sample analysed following a plasma sample spiked with a high concentration of all six analytes. To assess carry-over effects doxorubicin (DOX) and doxorubicinol (DOXol) were added to the high concentration samples to a concentration of 1000 µg·L^{-1} and doxorubicinone (DOXone), doxorubicinolone (DOXolone), 7-deoxydoxorubicinone (7-DeoxyDOXone) and 7-deoxydoxorubicinolone (7-DeoxyDOXolone) were added to a concentration of 100 µg·L^{-1}. The internal standard epirubicin (Epi) was added to a concentration of 40 µg·L^{-1}. Analyses were performed in triplicate.

Table S1: Summarized results from five calibration curves. Shown are mean values of the back calculated concentrations of the calibration standards together with the calculated mean accuracy values and % CV.

**Analyte**		**Nominal concentration [µg·L^{-1}]**										
		**1**	**2**	**5**	**10**	**25**	**50**	**100**	**250**	**500**	**750**	**1000**
**Doxorubicin**	**Mean [µg·L^{-1}]**	-	2.03	4.88	10.0	24.5	47.4	-	246	496	801	1053
	**SD [µg·L^{-1}]**	-	0.05	0.21	0.29	0.34	1.28	-	10.4	19.5	26.9	7.42
	**Accuracy [%]**	-	102	97.6	100	98.1	94.8	-	98.3	99.2	107	105
	**CV [%]**	-	2.3	4.4	2.9	1.4	2.7	-	4.2	3.9	3.4	0.7
**Doxorubicinol**	**Mean [µg·L^{-1}]**	-	2.03	4.88	9.91	25.1	47.4	102	258	523	-	-
	**SD [µg·L^{-1}]**	-	0.07	0.33	0.54	0.76	2.26	4.53	21.1	43.4	-	-
	**Accuracy [%]**	-	102	97.6	99.1	100	94.8	102	103	105	-	-
	**CV [%]**	-	3.3	6.7	5.4	3.0	4.8	4.5	8.2	8.3	-	-
**Doxorubicinone**	**Mean [µg·L^{-1}]**	1.03	1.95	5.18	10.1	24.5	50.9	99.0	-	-	-	-
	**SD [µg·L^{-1}]**	0.06	0.20	0.65	0.66	1.29	3.28	6.92	-	-	-	-
	**Accuracy [%]**	103	97.7	104	101	98.1	102	99.0	-	-	-	-
	**CV [%]**	6.1	10.1	12.5	6.5	5.3	6.4	7.0	-	-	-	-
**Doxorubicinolone**	**Mean [µg·L^{-1}]**	1.02	1.96	5.23	10.1	24.4	50.7	99.1	-	-	-	-
	**SD [µg·L^{-1}]**	0.05	0.19	0.66	0.68	1.18	3.26	6.75	-	-	-	-
	**Accuracy [%]**	102	97.9	105	101	97.6	101	99.1	-	-	-	-
	**CV [%]**	5.2	9.7	12.7	6.8	4.9	6.4	6.8	-	-	-	-
**7-Deoxydoxorubicinone**	**Mean [µg·L^{-1}]**	1.02	1.99	5.16	10.2	24.3	50.9	98.8	-	-	-	-
	**SD [µg·L^{-1}]**	0.06	0.21	0.68	0.70	1.19	3.19	7.96	-	-	-	-
	**Accuracy [%]**	102	99.6	103	102	97.1	102	98.8	-	-	-	-
	**CV [%]**	5.5	10.5	13.2	6.8	4.9	6.3	8.1	-	-	-	-
**7-Deoxydoxorubicinolone**	**Mean [µg·L^{-1}]**	1.03	1.97	5.15	10.1	24.4	51.0	99.2	-	-	-	-
	**SD [µg·L^{-1}]**	0.06	0.21	0.65	0.67	1.17	2.86	7.20	-	-	-	-
	**Accuracy [%]**	103	98.3	103	101	97.8	102	99.2	-	-	-	-
	**CV [%]**	6.2	10.9	12.6	6.6	4.8	5.6	7.3	-	-	-	-

SD, standard deviation; CV, coefficient of variation

Table S2: Results of the analysis of within-run and between-run accuracy and precision.

		Within-run (n=5)			Between-run (n=5)		
	**Nominal concentration [µg·L^{-1}]**	**Concentration [µg·L^{-1}] (mean + SD)**	**Accuracy [%]**	**CV [%]**	**Concentration [µg·L^{-1}] (mean + SD)**	**Accuracy [%]**	**CV [%]**
**Doxorubicin**	2	2.07 + 0.05	103.6	2.2	2.13 + 0.17	106.7	7.9
	5	5.01 + 0.07	100.3	1.4	5.08 + 0.25	101.7	5.0
	400	440 + 8.40	110.1	1.9	425 + 17.7	106.3	4.2
	750	858 + 31.6	114.4	3.7	787 + 41.2	104.9	5.2
**Doxorubicinol**	2	2.07 + 0.04	103.5	1.8	2.12 + 0.11	106.1	5.3
	5	5.07 + 0.20	101.4	4.0	5.24 + 0.23	104.8	4.3
	250	267 + 11.1	106.8	4.2	248 + 17.3	99.0	7.0
	400	458 + 22.2	114.6	4.8	440 + 18.8	110.1	4.3
**Doxorubicinone**	1	1.03 + 0.02	103.2	1.9	0.97 + 0.05	97.0	5.6
	3	2.97 + 0.15	99.1	5.1	2.83 + 0.07	94.2	2.4
	50	52.6 + 1.74	105.3	3.3	50.8 + 1.96	101.7	3.8
	75	77.7 + 4.55	103.6	5.8	75.7 + 5.73	101.0	7.6
**Doxorubicinolone**	1	1.02 + 0.01	102.3	0.7	0.95 + 0.06	95.2	5.8
	3	2.93 + 0.15	97.6	5.0	2.81 + 0.09	93.5	3.4
	50	52.9 + 1.48	105.8	2.8	51.2 + 2.41	102.4	4.7
	75	77.1 + 4.21	102.8	5.5	75.0 + 6.43	100.0	8.6
**7-Deoxydoxorubicinone**	1	1.02 + 0.01	101.6	1.1	0.95 + 0.05	94.8	5.6
	3	2.95 + 0.17	98.3	5.9	2.79 + 0.09	93.0	3.2
	50	52.0 + 1.76	104.0	3.4	50.5 + 2.27	101.1	4.5
	75	77.6 + 4.76	103.4	6.1	75.3 + 5.82	100.5	7.7
**7-Deoxydoxorubicinolone**	1	1.04 + 0.02	104.4	1.5	0.96 + 0.08	95.6	8.3
	3	2.96 + 0.16	98.8	5.3	2.80 + 0.08	93.4	3.0
	50	53.7 + 2.28	107.4	4.2	52.0 + 2.88	104.0	5.5
	75	77.0 + 4.63	102.6	6.0	74.7 + 5.89	99.6	7.9

SD, standard deviation; CV, coefficient of variation

Table S3: Short-term stability of the aglycones in plasma after 24 h at room temperature.

Analyte	Nominal concentration [µg·L^{-1}]	Concentration [µg·L^{-1}] (mean ± SD)	Accuracy [%]	CV [%]
**Doxorubicinone**	3	2.64 ± 0.32	88.0	12.2
	75	65.3 ± 4.70	87.1	7.2
**Doxorubicinolone**	3	3.17 ± 0.37	106	11.8
	75	76.5 ± 5.24	102	6.9
**7-Deoxydoxorubicinone**	3	3.10 ± 0.36	104	11.6
	75	75.7 ± 5.03	101	6.6
**7-Deoxydoxorubicinolone**	3	3.21 ± 0.37	107	11.5
	75	77.3 ± 5.27	103	6.8

SD, standard deviation; CV, coefficient of variation

Table S4: Long-term stability of the aglycones in plasma after 12 weeks at -20°C and -80°C.

		**Stability at -20°C**			**Stability at -80°C**		
**Analyte**	**Nominal concentration [µg·L^{-1}]**	**Concentration [µg·L^{-1}] (mean ± SD)**	**Accuracy [%]**	**CV [%]**	**Concentration [µg·L^{-1}] (mean ± SD)**	**Accuracy [%]**	**CV [%]**
**Doxorubicinone**	3	2.85 ± 0.24	95.0	8.4	2.84 ± 0.39	94.7	13.8
	75	67.5 ± 4.07	90.0	6.0	75.1 ± 0.94	100	1.2
**Doxorubicinolone**	3	2.73 ± 0.24	91.1	8.9	2.59 ± 0.21	86.4	8.3
	75	65.6 ± 4.05	87.5	6.2	74.9 ± 1.28	99.9	1.7

Table S4: Long-term stability of the aglycones in plasma after 12 weeks at -20°C and -80°C (continued).

**7-Deoxydoxorubicinone**	3	2.81 ± 0.24	93.6	8.5	2.74 ± 0.36	91.4	13.0
	75	65.8 ± 4.09	87.8	6.2	69.9 ± 0.91	93.2	1.3
**7-Deoxydoxorubicinolone**	3	2.76 ± 0.24	91.9	8.6	2.59 ± 0.26	86.3	10.2
	75	66.3 ± 3.94	88.3	6.0	73.7 ± 2.28	98.3	3.1

SD, standard deviation; CV, coefficient of variation

Table S5: Freeze-thaw stability of the aglycones after three cycles. Samples were frozen at -80°C.

Analyte	Nominal concentration [µg·L^{-1}]	Concentration [µg·L^{-1}] (mean ± SD)	Accuracy [%]	CV [%]
**Doxorubicinone**	3	2.75 ± 0.25	91.6	9.3
	75	74.1 ± 7.32	98.8	9.9
**Doxorubicinolone**	3	2.67 ± 0.23	89.0	8.7
	75	72.6 ± 7.00	96.8	9.6
**7-Deoxydoxorubicinone**	3	2.71 ± 0.26	90.2	9.6
	75	74.8 ± 7.53	99.7	10.1
**7-Deoxydoxorubicinolone**	3	2.74 ± 0.25	91.2	9.1
	75	74.5 ± 7.27	99.3	9.8

SD, standard deviation; CV, coefficient of variation

Table S6: Stability of the stock solutions after 24 h at room temperature.

Analyte	Nominal concentration [µg·L^{-1}]	Concentration [µg·L^{-1}] (mean ± SD)	Accuracy [%]	CV [%]
**Doxorubicinone**	100	104 ± 1.75	104	1.7
**Doxorubicinolone**	100	99.6 ± 1.56	99.6	1.6
**7-Deoxydoxorubicinone**	100	98.3 ± 1.53	98.3	1.6
**7-Deoxydoxorubicinolone**	100	98.0 ± 1.49	98.0	1.5

SD, standard deviation; CV, coefficient of variation

Table S7: Stability of processed and extracted samples after 24 h at -20°C.

Analyte	Nominal concentration [µg·L^{-1}]	Concentration [µg·L^{-1}] (mean ± SD)	Accuracy [%]	CV [%]
**Doxorubicinone**	3	2.94 ± 0.18	97.9	6.1
	75	76.5 ± 1.45	102	1.9
**Doxorubicinolone**	3	2.82 ± 0.17	93.9	6.0
	75	73.0 ± 1.56	97.3	2.1
**7-Deoxydoxorubicinone**	3	2.89 ± 0.17	96.2	5.9
	75	74.5 ± 1.57	99.3	2.1
**7-Deoxydoxorubicinolone**	3	2.94 ± 0.18	98.0	6.2
	75	76.0 ± 1.44	101	1.9

SD, standard deviation; CV, coefficient of variation

Table S8: Autosampler stability of processed samples after 24 h at 4°C.

Analyte	Nominal concentration [µg·L^{-1}]	Concentration [µg·L^{-1}] (mean ± SD)	Accuracy [%]	CV [%]
**Doxorubicinone**	3	2.93 ± 0.09	97.6	3.0
	75	79.0 ± 4.19	105	5.3
**Doxorubicinolone**	3	2.88 ± 0.10	96.1	3.4
	75	77.8 ± 4.24	104	5.4
**7-Deoxydoxorubicinone**	3	2.90 ± 0.10	96.6	3.4
	75	78.5 ± 3.69	105	4.7
**7-Deoxydoxorubicinolone**	3	2.88 ± 0.10	96.0	3.4
	75	77.8 ± 4.18	104	5.4

SD, standard deviation; CV, coefficient of variation

References

1. European Medicines Agency (2011) Guideline on bioanalytical method validation. EMEA/CHMP/EWP/192217/2009 Rev. 1 Corr. 2**
